# Age-Related Alterations of White Matter Integrity in Adolescents and Young Adults With Bipolar Disorder

**DOI:** 10.3389/fpsyt.2019.01010

**Published:** 2020-01-28

**Authors:** Sihua Ren, Miao Chang, Zhiyang Yin, Ruiqi Feng, Yange Wei, Jia Duan, Xiaowei Jiang, Shengnan Wei, Yanqing Tang, Fei Wang, Songbai Li

**Affiliations:** ^1^Department of Radiology, The First Affiliated Hospital of China Medical University, Shenyang, China; ^2^Brain Function Research Section, The First Affiliated Hospital of China Medical University, Shenyang, China; ^3^Department of Psychiatry, The First Affiliated Hospital of China Medical University, Shenyang, China

**Keywords:** bipolar disorder, diffusion tensor imaging, fractional anisotropy, white matter, neurodevelopment

## Abstract

**Background:**

Alterations of white matter integrity during adolescence/young adulthood may contribute to the neurodevelopmental pathophysiology of bipolar disorder (BD), but it remains unknown how white matter integrity changes in BD patients during this critical period of brain development. In the present study, we aimed to identify possible age-associated alterations of white matter integrity in adolescents and young adults with BD across the age range of 13–30 years.

**Methods:**

We divided the participants into two groups by age as follows: adolescent group involving individuals of 13–21 years old (39 patients with BD and 39 healthy controls) and young adult group involving individuals of 22–30 years old (47 patients with BD and 47 healthy controls). Diffusion tensor imaging (DTI) was performed in all participants to assess white matter integrity.

**Results:**

In the adolescent group, compared to those of healthy controls, fractional anisotropy (FA) values were significantly lower in BD patients in the left inferior longitudinal fasciculus, splenium of the corpus callosum and posterior thalamic radiation. In the young adult group, BD patients showed significantly decreased FA values in the bilateral uncinate fasciculus, genu of the corpus callosum, right anterior limb of internal capsule and fornix compared to healthy controls. White matter impairments changed from the posterior brain to the anterior brain representing a back-to-front spatiotemporal directionality in an age-related pattern.

**Conclusions:**

Our findings provide neuroimaging evidence supporting a back-to-front spatiotemporal directionality of the altered development of white matter integrity associated with age in BD patients during adolescence/young adulthood.

## Introduction

An increasing number of studies using magnetic resonance imaging (MRI) have strongly suggested that changes in white matter (WM) may underline the disruption of the normal interactions between brain regions seen in bipolar disorder (BD) patients, thus making it possible to use neuroimaging evidence to delineate pathophysiological mechanism of BD (1–4). To measure the alterations of WM in BD patients, diffusion tensor imaging (DTI) has been widely used to assess WM integrity as fractional anisotropy (FA) obtained by DTI is generally considered a reliable index of axonal integrity and low FA values are generally related to structural damage of WM or demyelination (5, 6). Although WM impairments have been demonstrated in BD patients using DTI, the findings appeared to be inconsistent (7–12). Adolescence/young adulthood is an important period for the development of WM during which FA values tend to increase and such an increase in FA value may represent the continued myelination and thickening of the diameters of fiber tracts (13–16). During brain maturation, myelination follows a back-to-front spatiotemporal directionality and lower-order brain areas, such as sensorimotor regions, mature earlier than higher-order brain areas that are associated with emotional and cognitive functions, such as the frontal lobes (17). Deviations in the typical pattern of normal WM development may lead to the disruption of normal neural connectivity, resulting in the onset and clinical symptoms frequently manifested in BD patients (18–21).

A number of DTI studies have already found alterations of WM integrity in BD patients during adolescence/young adulthood, however, it remains unknown how WM integrity changes in BD patients during this period. A previous study showed that age-associated changes in WM integrity followed a nonlinear trajectory in the corpus callosum (CC) in BD patients across the age range of 9–62 years (21). While greater age-related alterations of WM integrity were demonstrated in BD patients beginning in the second decade of life in the splenium of corpus callosum (SCC), this effect was more evident among BD patients beginning in the third decade of life in the genu of corpus callosum (GCC). The abnormal development of WM in the CC may lead to alterations of inter-hemispheric communication. Another longitudinal study suggested that there was an absence in the expected increase of FA values in the uncinate fasciculus (UF) in adolescents and adults with BD (4), suggesting that the changes in structural development of WM in the UF could link to the abnormal neurodevelopment of BD, causing the emotional instability. Taken together, these findings suggested that the abnormal development of WM may contribute to the pathophysiology of BD.

In this study, we used a voxel-based analysis of DTI to examine the WM integrity of all participants across the age range of 13–30 years by measuring FA values. fMRI findings suggested that the mean brain age achieving a maximum level of maturity is 22.3 years old and it may represent a vital period of time with a great venerability in the development of brain prior to the maturity (22, 23). Additionally, Nurnberger et al. found that clinical assessments performing on offspring aged 12–21 years from families with a proband with BD may identify those prone to the onset of major affective disorders during adolescence (24). The findings indicated that BD patients of 12–21 years old present some clinical symptoms that differ from those of BD patients older than 21 years. Furthermore, a previous study examining the contributions of age at onset and childhood psychopathology in psychotic BD patients demonstrated that onset of BD at an early age prior to twenty was generally associated with poorer functional and clinical outcomes, suggesting that differences in brain development underline the mechanisms in BD during adolescence and adulthood (25). Therefore, we divided the participants into two groups by age 21 as follows: adolescent group involving individuals of 13–21 years old and young adult group involving individuals of 22-30 years old. By comparing differences in the WM between BD patients and health controls (HC), we aimed to investigate the age-associated alterations of the brain during adolescent/young adulthood in BD patients and to delineate the neurodevelopmental pathophysiology of BD.

## Materials and Methods

### Participants

There were 86 individuals with BD and 86 HC involved in the present study. All participants were divided into two groups by age as follows: adolescent group with the age range of 13–21 years and young adult group with the age range of 22–30 years. The numbers of both BD patients and HC in each group were 39 and 47, respectively. The age distribution showed as following: there were 10 individuals of 13–15 years old (5 BD patients, 5 HC), 38 individuals of 16–18 years old (17 BD patients, 21 HC), 30 individuals of 19–21 years old (17 BD patients, 13 HC), 30 individuals of 22–24 years old (15 BD patients, 15 HC), 36 individuals of 25–27 years old (18 BD patients, 18 HC), and 28 individuals of 28–30 years old (14 BD patients, 14 HC), respectively. BD patients were recruited from the outpatient services at the Department of Psychiatry, First Affiliated Hospital of China Medical University and Mental Health Center of Shenyang, while HC were recruited from the local community by advertisements matched for gender and age. After receiving a detailed description of the study, all participants (or parents or guardian for those under 18 years old) provided written informed consent. The study was approved by the Medical Research Ethics Committee of the China Medical University in accordance with the Declaration of Helsinki. Consensus was made between two trained psychiatrists to confirm the presence or absence of Axis I diagnoses using the following criteria: the Structured Clinical Interview for Diagnostic and Statistical Manual of Mental Disorders, Fourth Edition (DSM-IV) Axis I Disorders for participants of 18 years old or older and the Schedule for Affective Disorders and Schizophrenia for School-Age Children-present and Lifetime Version for participants younger than 18 years old. BD participants met DSM-IV diagnostic criteria for BD without any other Axis I disorders. No presence of any other DSM-IV Axis I disorders was confirmed in BD patients or their first-degree family members. Individuals were excluded from participation based on the following criteria: 1) general contraindications for MRI, 2) history of substance/alcohol abuse or dependence, 3) history of head trauma with loss of consciousness for ≥ 5 min or any neurological disorder, and 4) concomitant major medical disorder. For all participants, mania scores were assessed using the Young Mania Rating Scale (YMRS), depression scores were assessed using the 17-item Hamilton Depression Rating Scale (HAMD-17), and anxiety scores were assessed using the Hamilton Anxiety Scale (HAMA). Details of age of onset, first episode, and duration of illness in BD patients were obtained. Considering mood states, in the adolescent group, there were 11 BD patients in a stable state, 16 BD patients in a depressed state and 9 BD patients in a manic state. In the young adult group, there were 13 BD patients in a stable state, 19 BD patients in a depressed state and 10 BD patients in a manic state. Considering medication status, in the adolescent group, there were 14 BD patients on mood stabilizers, 14 BD patients on antipsychotics, 9 BD patients on antidepressants, and 16 BD patients without medication. In the young adult group, there were 25 BD patients on mood stabilizers, 21 BD patients on antipsychotics, 21 BD patients on antidepressants, and 16 BD patients without medication.

### DTI Acquisition and Processing

To obtain MRI data, a GE Signa HDX 3.0T MRI scanner was used with a standard head coil at this hospital. Head motion was minimized with restraining foam pads. To acquire DTI, spin-echo planar imaging sequences were used with the following parameters: TR = 17000 ms, TE = 85.4 ms, FOV = 24 cm × 24 cm, imaging matrix = 120 × 120, 65 contiguous axial slices of 2 mm without gap, 25 non-collinear directions (b = 1000 s/mm^2^), axial acquisition without diffusion weighting (b = 0), and voxel size = 2.0 mm^3^.

The acquired images were processed with PANDA software (http://www.nitrc.org/projects/panda). To transform individual FA images of native space to a standard Montreal Neurological Institute (MNI) space, spatial normalization (voxel size = 1 mm × 1 mm × 1 mm) was used after motion and eddy-current correction were applied. Finally, to reduce misalignment and noise, the FA images were smoothed by a 6-mm Gaussian kernel.

### Statistical Analysis

We used student *t* test and χ^2^ test to compare demographic data and the YMRS, HAMD-17, HAMA scores between BD patients and HC in the adolescent and young adult groups. We also used χ^2^ test to compare the composition of different mood states between adolescents with BD and young adults with BD. The *t* test was also employed to determine if there was a significant difference in FA values between BD patients and controls separately in the adolescent and young adult groups. The voxel-level inference of *p* < 0.005 with Gaussian random field (GRF) correction for cluster-level inference of *p* < 0.05 was used for statistical inference in DTI images. Moreover, FA values that were extracted from the regions showing significant differences between BD patients and HC were used for partial correlation analyses to explore the clinical factors including the YMRS, HAMD-17, and HAMA scores. To rule out the possible interference on the dynamics of FA performance in the medicated patients, the difference in FA values was further determined by t test in the specified brain regions between medication-free BD patients and HC. For multiple comparisons, statistical significance was set at *p* < 0.05 corrected by Bonferroni. We also performed analysis of variance (ANOVA) by using group (BD patients vs HC) and age (adolescents versus young adults) as between subject factors to investigate the group × age interaction in the brain regions where the significant differences in FA values between BD patients and HC in the adolescent and young adult groups were observed in the above *t* tests. Post hoc analyses were used to determine the extent of alterations in FA values among adolescents with BD, healthy adolescents, young adults with BD and healthy young adults.

## Results

Overall, there were no significant differences in age, gender and handedness between BD patients and HC in adolescent and young adult groups. Additionally, there was no significant difference in the composition of different mood states between adolescents with BD and young adults with BD. However, BD patients had significantly greater levels of manic depression and anxiety as measured by the YMRS, HAMD-17, and HAMA compared to those of controls (**Table 1**).

**Table 1:** Demographic and clinical characteristics of study participants.

Characteristics	Age of 13–21 years old		*t*/χ^2^ *P*	Age of 22–30 years old		*t*/χ^2^ *P*
	BD (n = 39)	HC(n = 39)		BD (n = 47)	HC (n = 47)	
	Mean (SD)	Mean (SD)		Mean (SD)	Mean (SD)	
Age at scan (years)	17.90 (2.19)	17.85 (2.22)	0.103 0.918	25.87 (2.75)	25.72 (2.56)	0.272 0.786
Gender (male/female)	14/25	16/23	0.217 0.642	24/23	21/26	0.384 0.536
Handedness (R/L/MIX)	36/1/2	37/0/0	2.963 0.227	46/0/0	44/0/2	2.044 0.153
First episode (yes/no)	25/14	—	—	24/21	—	—
Age of onset (years)	15.6 (2.08)	—	—	22.3 (4.88)	—	—
State (stable/depressed/manic)	11/16/9	—	—	13/19/10	—	—
Medication (yes/no)	23/16	—	—	31/16	—	—
Duration (months)	22.42 (20.17)	—	—	33.52 (36.27)	—	—
YMRS	n = 39	n = 31		n = 45	n = 42	
	7.87 (10.29)	0.07 (0.25)	4.737< 0.001	8.73 (10.92)	0.12 (0.33)	5.289< 0.001
HAMD-17	n = 38	n = 31		n = 47	n = 43	
	11.7 (9.21)	1.65 (1.78)	6.584< 0.001	12.85 (11.03)	0.81 (1.20)	7.433< 0.001
HAMA	n = 36	n = 30		n = 45	n = 30	
	8.42 (7.30)	0.57 (1.07)	6.371< 0.001	9.78 (10.75)	0.47 (1.10)	5.779 < 0.001

SD, standard deviation; BD, bipolar disorder; HC, healthy controls; YMRS, Young Mania Rating Scale; HAMD, Hamilton Depression Rating Scale; HAMA, Hamilton Anxiety Scale.

**Figure 1:**
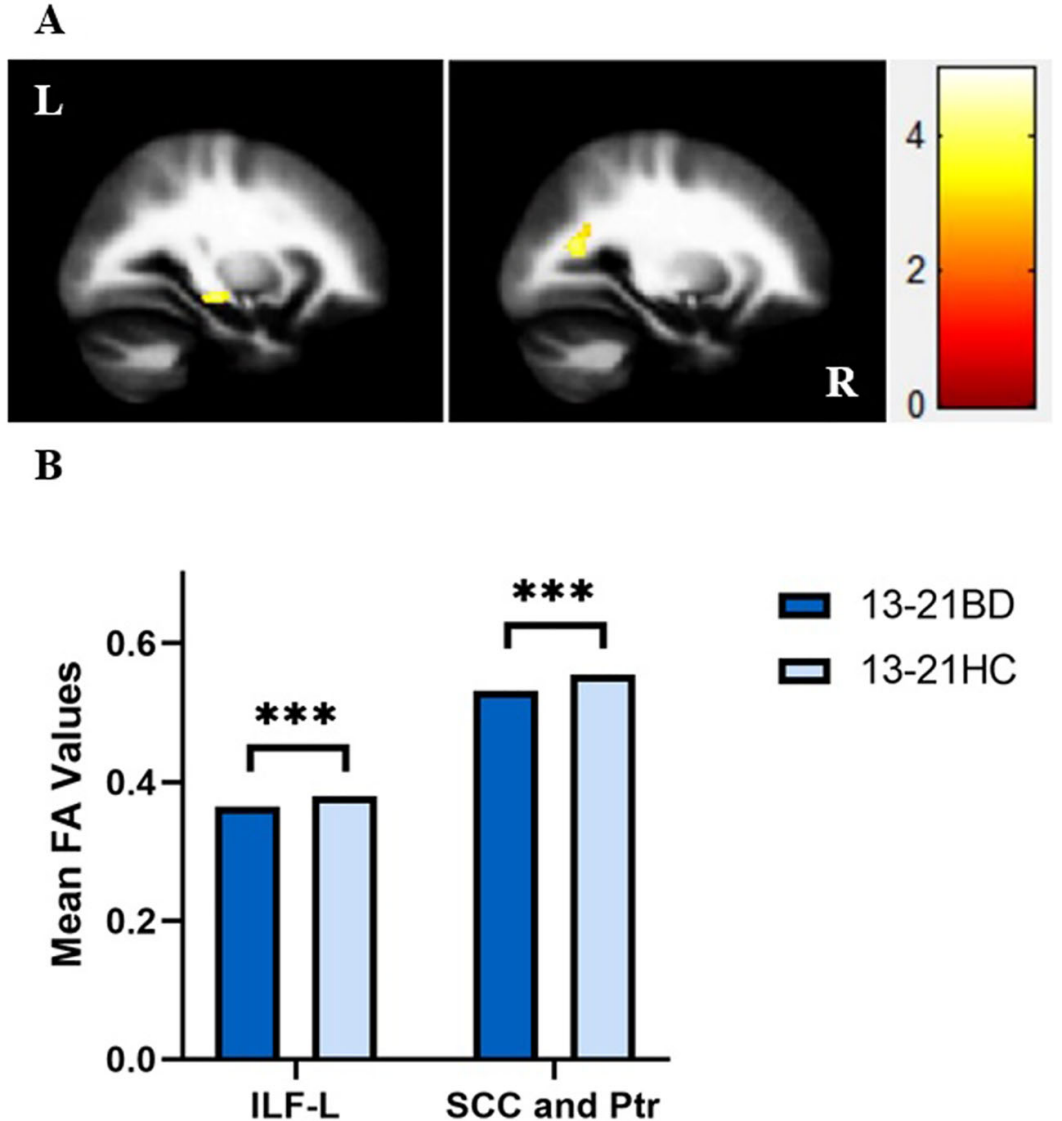
**(A)** Sagittal views showing significant differences in fractional anisotropy (FA) values in the left inferior longitudinal fasciculus (ILF), splenium of corpus callosum (SCC) and posterior thalamic radiation (Ptr) between bipolar disorder (BD) patients and healthy controls (HC) in the adolescent group from left to right as follows: (1) left ILF, and (2) SCC and Ptr [Montreal Neurological Institute (MNI) coordinates, respectively: x = -28 mm, y = -22 mm, z = -10 mm, 124 voxels, T = 4.11; x = -26 mm, y = -62 mm, z = 18 mm, 122 voxels, T = 4.25, *p* < 0.005 with Gaussian random field (GRF) correction]. **(B)** FA values in white matter fibers extracted from the left ILF, SCC and Ptr in BD patients and HC in the adolescent group. ***p < 0.001. ILF-L, left inferior longitudinal fasciculus; L, left brain; R, right brain. Color bars represent T values.

**Figure 2:**
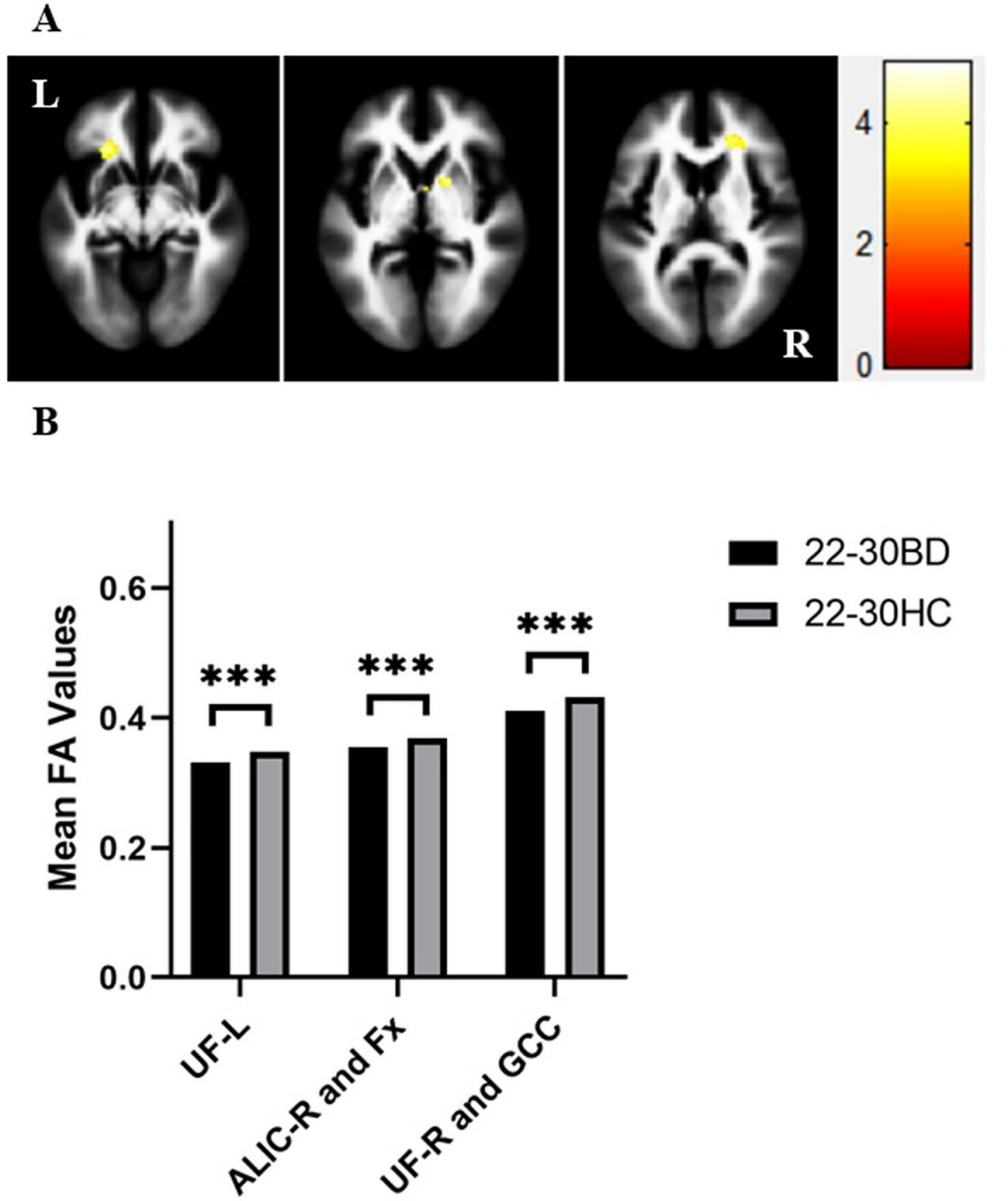
**(A)** Axial views showing significant differences in fractional anisotropy (FA) values in the bilateral uncinate fasciculus (UF), genu of corpus callosum (GCC), right anterior limb of internal capsule (ALIC) and fornix (Fx) between bipolar disorder (BD) patients and healthy controls (HC) in the young adult group from left to right as follows: (1) left UF, (2) right ALIC and Fx, and (3) right UF and GCC [Montreal Neurological Institute (MNI) coordinates, respectively: x = -24 mm, y = 28 mm, z = -8 mm, 138 voxels, T = 4.54; x = 16 mm, y = 6 mm, z = 0 mm, 84 voxels, T = 4.31; x = 20 mm, y = 34 mm, z = 10 mm, 181 voxels, T = 4.11, *p <* 0.005 with Gaussian random field (GRF) correction]. **(B)** FA values in white matter fibers extracted from the bilateral UF, GCC, right ALIC and Fx in BD patients and HC in the young adult group. ***p < 0.001. UF-L, left uncinate fasciculus; ALIC-R, right anterior limb of the internal capsule; UF-R, right uncinate fasciculus; L, left brain; R, right brain. Color bars represent T values.

In our present study, adolescents with BD showed significantly decreased FA values compared to those of healthy adolescents in the distinct brain regions as follows:1) left inferior longitudinal fasciculus (ILF), and 2) SCC and posterior thalamic radiation (MNI coordinates, respectively: x = -28 mm, y = -22 mm, z = -10 mm, 124 voxels, T = 4.11; x = -26 mm, y = -62 mm, z = 18 mm, 122 voxels, T = 4.25; *p <* 0.005 corrected by GRF) (**Figure 1**). Compared to those of HC, FA values were significant lower in BD patients in the young adult group in the following brain areas: 1) left UF, 2) right anterior limb of internal capsule (ALIC) and fornix, and 3) right UF and GCC (MNI coordinates, respectively: x = -24 mm, y = 28 mm, z = -8 mm, 138 voxels, T = 4.54; x = 16 mm, y = 6 mm, z = 0 mm, 84 voxels, T = 4.31; x = 20 mm, y = 34 mm, z = 10 mm, 181 voxels, T = 4.11; *p <* 0.005 corrected by GRF (**Figure 2**). There were no significant correlations between mean FA values and the YMRS, HAMD-17, and HAMA scores (*p* > 0.05, Bonferroni corrected for six times) in the adolescent group. In the young adult group, there were no significant correlations between mean FA values and the YMRS, HAMD-17, and HAMA scores (*p* > 0.05, Bonferroni corrected for nine times). Regardless of age, the medication-free patients showed significant differences in FA values compared to HC (adolescents with BD vs healthy adolescents: *p* < 0.05, Bonferroni corrected for two times; young adults with BD versus healthy young adults: *p* < 0.05, Bonferroni corrected for three times, respectively).

**Figure 3:**
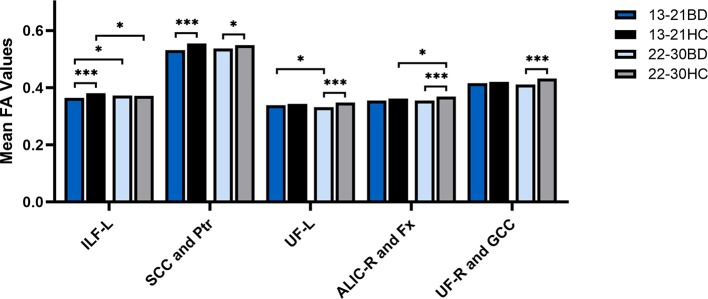
FA values in white matter fibers focusing on the group × age interaction extracted from the left inferior longitudinal fasciculus (ILF), splenium of corpus callosum (SCC), posterior thalamic radiation (Ptr), bilateral uncinate fasciculus (UF), genu of corpus callosum (GCC), right anterior limb of internal capsule (ALIC) and fornix (Fx) in adolescents with bipolar disorder (BD), healthy adolescents, young adults with BD and healthy young adults. *p < 0.05, ***p < 0.001. ILF-L, left inferior longitudinal fasciculus; UF-L, left uncinate fasciculus; ALIC-R, right anterior limb of the internal capsule; UF-R, right uncinate fasciculus; L, left brain; R, right brain. Color bars represent T values.

We further determined if there was a group × age interaction in the brain regions where the significant differences in FA values between BD patients and HC in the adolescent and young adult groups were observed in the above *t* tests. There was a significant group × age interaction in the left ILF, SCC, posterior thalamic radiation, left UF, right ALIC, and fornix (all *p* < 0.05). The *post hoc* analyses demonstrated that the larger contribution to the group × age interaction was derived mainly from the decreased FA values in the left ILF, SCC and posterior thalamic radiation in adolescents with BD compared to healthy adolescents (all *p* < 0.001). Compared to those of HC, FA values in the left ILF showed no significant differences in young adults with BD (*p* > 0.05) and greater extent of changes in the mean FA values was found in the SCC and posterior thalamic radiation in adolescents with BD (*p* < 0.001) compared to young adults with BD (*p* < 0.05). Additionally, there were significant lower FA values in the left UF, right ALIC, and fornix indicating the larger contribution to the interaction between group and age in young adults with BD compared to HC (all *p* < 0.001), and FA values in the above brain regions showed no significant differences between adolescents with BD and healthy adolescents, although the group × age interaction in the right UF and GCC was not significant (**Figure 3**).

## Discussion

To our knowledge, this is the first report to demonstrate the age-associated alterations of WM integrity following a back-to-front spatiotemporal directionality in BD patients during adolescence/young adulthood. Our findings yielded from DTI analysis indicated that the adolescent BD patients showed significantly lower FA values in the left ILF, SCC, and posterior thalamic radiation compared to those of HC. While in the young adult group, FA values were significantly decreased in the bilateral uncinate fasciculus, genu of the corpus callosum, right anterior limb of internal capsule, and fornix compared to those of HC. The disruptions of neural connectivity within WM apparently changed from the posterior brain to the anterior brain in an age-dependent manner. Our DTI findings further revealed that there were alternations in anatomical connectivity in neural microstructures of WM in BD patients in all age groups and highlighted the importance of considering WM integrity in the neurodevelopmental pathophysiology of BD.

One of critical findings of our study was that development of abnormal WM integrity in BD patients follows an apparent back-to-front spatiotemporal directionality of WM and it was also the age-dependent. Interestingly a similar directionality of the normal development of the brain has been reported in the previous findings (14, 17, 26). Studies using structural MRI to investigate gray matter density showed that the decline in gray matter density and increased myelination have been companied with continued normal brain growth until the age of 30 years old. The trajectory of maturational and aging changed heterogeneously over the cortex as follows: cortices such as the visual and auditory cortex myelinated earlier and showed a more linear pattern of aging compared to the frontal cortex. Another study investigating cortical development using MRI demonstrated that lower-order brain areas such as sensorimotor regions matured earlier than higher-order brain areas such as the frontal cortex. Furthermore, a previous study using DTI to investigate WM integrity also suggested a back-to-front spatiotemporal development of the normal brain (27). The findings indicated that the more rostral portions of the CC and internal capsule began the maturation process latter than the caudal regions. Interestingly, the back-to-front changes of brain were also found in BD patients demonstrated in previous studies in which either fMRI or DTI was used as a research tool (28, 29). fMRI studies investigating connectivity and network of brain showed that the abnormal input from lower-order brain regions may affect higher-order brain regions and cause dysfunction within neural circuits, which may act on sensory processing circuits later and finally lead to the clinical symptoms of BD. While a DTI study demonstrated that greater age-associated changes in WM integrity in BD patients began in the second decade of life within the SCC and began in the third decade of life within the GCC (21), which were consistent with our findings. However, a recent study showed that there was a strong correlational relationship between the extent of quadratic measurement difference and peak maturational timing (30). The findings of the study supported the last-in-first-out petrogenesis hypothesis of aging (31). To this regard, more longitudinal studies are needed to explore the neurodevelopmental pathophysiology of BD.

As suggested in our findings, WM disruptions changed from the posterior brain involving primary cortex to the anterior brain involving high-order cortex in BD patients during adolescence/young adulthood. Alterations of WM in the back of the brain have been demonstrated to be related to the impairments of primary sensory processing by previous studies. For example, the ILF connects occipital and temporal lobes and relays information to the orbitofrontal region of the brain (32). Decrease in FA values in ILF and the changes of WM integrity were associated with the impairments of object recognition in children (33). Additionally, the decreased FA values in the posterior thalamic radiation in BD patients in the present study were almost identical to a previous study in which, using a visual backward masking task, the impairments of visual processing were associated with deficits in neural pathways involving posterior brain areas in BD patients (6, 34). As the alterations of WM were mainly confined in the front of the brain in young adults with BD as suggested by our findings, the disruptions of WM may lead to abnormal higher-order functions such as cognitive and emotional regulation. For example, the UF is crucial for communication between the amygdala and ventral prefrontal cortex and plays an important role in maintain emotional stability (4, 35–37). Decreased FA values found in the UF in BD patients could suggest there could be an abnormality in the perigenual anterior cingulate cortex-amygdala functional connection and the impairments of WM microneural connectivity may lead to the abnormality of emotional processing in BD (38). Additionally, the GCC, an area connecting the frontal cortices, participates in higher-order cognitive and emotional regulation (8, 39–44) and any deficits of prefrontal connectivity may cause clinical symptoms of BD such as altered responses to emotional stimuli (45, 46). Moreover, the ALIC may interconnects with higher-order brain regions such as prefrontal cortex and amygdala through thalamic nuclei and structural abnormalities in the ALIC and UF could cause psychotic symptoms and mood dysregulation including increased risk taking behaviors (47). Furthermore, the damage of the fornix was related to impaired amnesic function involved in the clinical manifestations of BD as the fornix serve as a major efferent fiber bundle from the hippocampus (48–50).

Moreover, there were several limitations in the present study. First, the study was cross-sectional and the potential alterations of WM integrity which may occur in the development of BD remain unknown. Further longitudinal studies may produce the validity of our findings supporting developmental changes are associated with the spectrum of BD. Second, the relative smaller sample size of this study prevents us to provide additional data critical for further delineating the neurodevelopmental pathophysiology of BD. Third, the results obtained from BD patients who received medications could complicate interpretations of the findings. However, regardless of their age, BD patients without medications showed significant differences in FA values when compared to HC and this finding was consistent with our previous findings. Further studies comprising of unmedicated patients could unambiguously exclude the effect of medication on FA values in the BD patients. Fourth, as the youngest patient in our study is only 13 years old and an age-appropriate standard template should be used in future study. Fifth, there was a partially overlapped age of onset in adolescents with BD and young adults with BD in our study. Patients in distinctive ages should be included in the further analysis in order to reduce the effect of the overlapped age. Finally, a paucity of evidence provided by DTI studies indicates that age around 21 could arguably be a pivotal timing for the development of BD, clearly, more longitudinal DTI studies are needed to confirm the hypothesis.

In summary, our study suggests that there are age-related alterations of WM integrity in adolescents and young adults with BD. A back-to-front spatiotemporal directionality of WM impairments share a similar directionality of the normal brain development. The findings highlight the importance of considering the neurodevelopmental pathophysiology of BD.

## Data Availability Statement

The datasets generated for this study are available on request to the corresponding authors.

## Ethics Statement

The studies involving human participants were reviewed and approved by the Ethics Committee of the First Affiliated Hospital of China Medical University. Written informed consent to participate in this study was provided by the participants’ legal guardian/next of kin or the participants themselves.

## Author Contributions

SR, YT, FW, and SL designed the study. MC, ZY, RF, YW, JD, XJ, and SW participated in data collection, preprocessing and analysis. All the authors were involved in data interpretation, writing, and manuscript preparation and approved the final manuscript.

## Funding

The study was funded by National Natural Science Foundation of China (81571311 to YT, 81571331 to FW), National Science Fund for Distinguished Young Scholars (81725005 to FW), National Key Research and Development Program (2018YFC1311604 to YT, 2016YFC1306900 to YT, 2016YFC0904300 to FW), National High Tech Development Plan (863) (2015AA020513 to FW), Liaoning Science and Technology Project (2015225018 to YT), Innovation Team Support Plan of Higher Education of Liaoning Province (LT2017007 to FW), Major Special Construction Plan of China Medical University (3110117059 to FW).

## Conflict of Interest

The authors declare that the research was conducted in the absence of any commercial or financial relationships that could be construed as a potential conflict of interest.

## Abbreviations

ALIC, anterior limb of internal capsule; ANOVA, analysis of variance; BD, bipolar disorder; CC, corpus callosum; DSM-IV, diagnostic and statistical manual of mental disorders, fourth edition; DTI, diffusion tensor imaging; FA, fractional anisotropy; fMRI, functional magnetic resonance imaging; GCC, genu of the corpus callosum; GRF, gaussian random field; HAMA, hamilton anxiety scale; HAMD-17, 17-item hamilton depression rating scale; HC, healthy controls; ILF, inferior longitudinal fasciculus; MNI, montreal neurological institute; MRI, magnetic resonance imaging; SCC, splenium of the corpus callosum; UF, uncinate fasciculus; WM, white matter; YMRS, young mania rating scale.
